# Clinical study on the effects of different cold compress methods and durations on postoperative complications following mandibular impacted third molar extraction

**DOI:** 10.3389/fsurg.2025.1627139

**Published:** 2025-11-24

**Authors:** Zhiwen Xie, Wenjuan Zhang, Tianxiang Du, Ying Wang, Jiantao Wang, Jinlu Li, Pengfei Qu

**Affiliations:** Department of Oral and Maxillofacial Surgery, The Second Hospital of Hebei Medical University, Shijiazhuang, Hebei, China

**Keywords:** clinical first-hand data, cold compress methods, cold compress duration, mandibular impacted third molar extraction, postoperative complications

## Abstract

**Background:**

To optimize the postoperative cold compress protocol for mandibular impacted third molar extraction.

**Methods:**

Subjects were randomly divided into two major groups. The first group compared continuous vs. intermittent cold compress application, while the second group evaluated the duration of cold compress therapy. Postoperative outcomes, including pain intensity, facial swelling, trismus, and wound hemorrhage, were systematically analyzed.

**Results:**

Continuous cold compress application within the first 6 h postoperatively demonstrated superior efficacy over intermittent application in alleviating pain, reducing swelling, improving mouth opening, and minimizing hemorrhage incidence. Cold compress application during postoperative D1 significantly controlled hemorrhage and mitigated acute pain. Prolonged therapy to D3 further enhanced facial edema reduction and trismus resolution.

**Conclusions:**

A protocol of continuous cold compress application for 6 h daily during the initial 3 postoperative days significantly reduces complications, offering optimal clinical outcomes.

## 1 Introduction

According to a recent meta-analysis, which was based on 98 studies (involving 183,828 subjects), the data on the prevalence of third molar impaction worldwide was obtained. Overall, the prevalence of impacted third molars was 36.9% per subject and 46.4% per tooth, with the highest rates in Asia (43.1%) and the lowest in Europe (24.5%) (1). The extraction of mandibular impacted third molars, involving osteotomy and mucoperiosteal flap reflection, frequently induces postoperative complications such as localized swelling, pain, wound hemorrhage, and trismus (2). The postoperative pain after mandibular impacted third molar extraction mainly results from tissue damage, inflammatory response and nerve compression (3). The mechanisms of postoperative swelling include increased vascular permeability, impaired lymphatic return and the effect of gravity (4). The mechanisms of trismus include muscle spasms, increased intra-articular pressure and neuromuscular coordination disorders (5). The mechanisms of postoperative hemorrhage include vascular injury, abnormal coagulation function and blood pressure fluctuations (6). These sequelae significantly impair patients' fundamental physiological functions, including mastication and phonation (7). Recently, numerous studies have been dedicated to exploring treatment methods for alleviating these postoperative sequelae. Motonobu et al. suggested that bilateral impacted mandibular third molar extractions under intravenous sedation could reduce the pain intensities and number of oral analgesic doses (8). Momeni et al. indicated that extraoral low-level laser therapy could decrease pain but not edema and trismus after surgical extraction of impacted mandibular third molars (9). Additionally, Soltaninia et al. indicated that mannitol infiltration significantly reduced postoperative pain and trismus in impacted third molar surgery (10). According to the “Postoperative Management Guidelines” released by the American Association of Oral and Maxillofacial Surgeons (AAOMS), the adjunctive treatment must meet the following conditions: it must be non-invasive, be able to directly alleviate acute reactions (such as pain and swelling), and work in synergy with the main surgical/drug treatment plan, but it should not replace the core treatment (such as tooth extraction, the use of antibiotics) (11). As a non-pharmacological intervention, cold compress therapy achieves symptom control through physical effects (vasoconstriction, inhibition of nerve conduction), without the burden of drug metabolism (12). It has been reported that using cooling therapy methods can significantly reduce postoperative swelling and pain of patients undergo third molar surgery (13). A study proposed by do Nascimento-Júnior et al. (14) suggested cryotherapy may have a small benefit in reducing pain after third-molar surgery, but it is not effective on facial swelling and trismus. Owing to the lack of standardization of cold application, effective evidence-based treatment protocols for cryotherapy after third-molar surgery still need to be established. Meanwhile, critical parameters in existing clinical guidelines—such as optimal duration, application frequency, and modality—remain inadequately standardized due to insufficient evidence-based medical consensus (15–17), resulting in substantial variability in clinical practice.

This study aims to establish first-hand clinical data through phased clinical trials to formulate evidence-based standardized protocols and advance postoperative management strategies following impacted molar extraction. This research breaks away from the simplistic “cold compress vs. no cold compress” comparison, and divides the cold compress methods into continuous cold compress (maintaining a constant low temperature for 6 h) and intermittent cold compress (applying cold compress for 30 min and then resting for 1 h, repeating this process for 6 h). This layered design directly addresses the clinical pain point of the lack of unified standards for “cold compress time and frequency”, providing an operational guideline for postoperative care. In addition, this study, through multi-dimensional assessment, not only revealed the causal relationship between cold compress methods and postoperative complications, but also promoted the development of postoperative care in oral and maxillofacial surgery towards the direction of “precision, individualization, and multimodality”, providing high-level evidence support for clinical practice.

## 2 Materials and methods

### 2.1 Overall study overview

This study was a comprehensive investigation encompassing four distinct sub-studies, each with its own specific objectives and endpoints. The overall study was conducted with approval from the Institutional Ethics Committee (Approval No.: 2024-R649), and written informed consent was obtained from all participants. The clinical registration number was ChiCTR2000035334.

### 2.2 Sub-study 1: survey study

#### 2.2.1 Study design

This was a cross-sectional survey study aimed at collecting patients' recommendations on the optimal cold compress duration for pain relief and quality of life improvement after mandibular impacted third molar extraction.

#### 2.2.2 Sample

The authors conducted a survey on 60 patients who had undergone mandibular impacted third molar extraction at the Department of Oral and Maxillofacial Surgery.

#### 2.2.3 Questionnaire design

A questionnaire was first designed, including patients' age, sex, action of postoperative cold compress, cold compress material, cold compress method and whether frostbite has occurred. The questionnaires were filled out based on the patients' conditions.

### 2.3 Sub-study 2: pilot study

#### 2.3.1 Study design

This was a pilot study designed to simulate real-world clinical scenarios and prevent cryotherapy-related adverse events. It also aimed to refine protocols for subsequent randomized controlled trials (RCTs).

#### 2.3.2 Sample

A total of 30 cases were involved in this pilot study. These patients were selected from those who had undergone mandibular impacted third molar extraction at the Department of Oral and Maxillofacial Surgery.

#### 2.3.3 Intervention and monitoring

Similar to the later RCTs, cold compress therapy was applied, and skin temperatures and safety were closely monitored during the process.

#### 2.3.4 Endpoints

The endpoints included the incidence of frostbite and other adverse events related to cold compress, as well as the feasibility of the cold compress protocols in a real-world setting. The findings were used to optimize the protocols for the subsequent RCTs.

### 2.4 Sub-study 3: study of intermittent vs. continuous cold compress

#### 2.4.1 Study design

This was a prospective randomized controlled clinical trial comparing the effects of intermittent and continuous cold compress on patients after mandibular impacted third molar extraction.

#### 2.4.2 Sample size calculation

A power analysis using the G*Power 3.1.9.7 software was conducted. Under the set *α* value of 0.05 and a 90% power analysis condition, each group (intervention and control) required 38 patients as the sample size.

#### 2.4.3 Randomization and blinding

The stratified randomization method was adopted, with the number of teeth extracted (one or two) as the stratification variable. After determining the stratification variables, a random allocation sequence for different stratifications (namely, extracting one tooth and extracting two teeth) was generated using a computer. To ensure the confidentiality of the allocation, a series of security measures were taken for the generated random allocation sequence. Each random allocation sequence within each stratum was given a consecutive number, and then the numbered sequences were placed separately into sealed and opaque envelopes. These envelopes were strictly kept before the inclusion of patients who met the inclusion criteria to prevent information leakage. During the patient recruitment process, when a patient met the inclusion criteria, their corresponding stratification group was determined based on the actual number of teeth extracted. Then, the opaque envelope corresponding to that stratification group was opened, and the patient was assigned to the appropriate group according to the random allocation sequence indicated in the envelope. Eventually, after all 76 patients who met the inclusion criteria completed the allocation, they were randomly and evenly divided into the control group and the intervention group, with 38 patients in each group.

#### 2.4.4 Inclusion and exclusion criteria

Inclusion Criteria: Patients meeting the indications for third molar extraction with no absolute contraindications; Diagnosis of mandibular impacted third molar confirmed by clinical oral examination and panoramic/x-ray imaging, requiring osteotomy, flap reflection, and removal of dental/bony obstructions during extraction; Surgical duration <2 h, with no intraoperative root fracture or adjacent tooth injury.

Exclusion Criteria: Allergy to local anesthetics (e.g., lidocaine) or use of postoperative analgesic interventions; Positive cold sensitivity test; History of diabetes mellitus or coagulation disorders; Peripheral circulatory insufficiency or sensory neuropathy affecting pain perception; Midway withdraw from the study.

#### 2.4.5 Surgical methods

All patients were advised to perform mandibular impacted third molar extraction after having breakfast in the morning. After the visit, panoramic x-rays were taken for all patients. Before the operation, the difficulty of the surgery and the risks during and after the procedure were analyzed. Each patient signed the informed consent form for tooth extraction at the Department of Oral and Maxillofacial Surgery, The Second Hospital of Hebei Medical University. All the surgeries were performed by the first author of this study alone. Routine disinfection, dressing, anesthesia, incision and flap lifting, and extraction of impacted teeth were carried out. All patients had panoramic x-rays taken after the surgery to confirm that there were no remaining tooth fragments or bone fragments. After the complete extraction of the teeth, the patients were instructed to take antibiotics including cefadroxil tablets (0.5 g/time, twice a day; CSPC Ouyi Pharmaceutical Co., Ltd) and ornidazole dispersible tablets (0.25 g/time, twice a day; HENAN TOPFOND PHARMACEUTICAL CO., LTD) orally for 3 days.

During each operation, 1–2 teeth were extracted and a unilateral approach was adopted. After the teeth on one side were extracted, factors such as the difficulty of the extraction, the patient's recovery condition, and overall health status were taken into consideration to determine the timing of extracting the teeth on the other side. It was usually recommended to wait 2 to 4 weeks, but the specific time was determined by the doctor based on the individual situation.

#### 2.4.6 Intervention protocols

Control group: Intermittent cold compress protocol: 30 min cold compress sessions alternating with 1 h intervals, repeated for 6 h.

Intervention group: Continuous cold compress applied uninterrupted for 6 h. Before implementing continuous cold compress, we conducted a comprehensive assessment of all the patients in this group, inquiring and recording their past medical history in detail, with particular attention to whether there were any dangerous conditions related to prolonged cold compress, such as local circulatory disorders (such as Raynaud's disease), abnormal skin sensation disorders, history of cold allergy, local skin damage or infection. After the assessment, it was found that all the patients included in the continuous cold compress group in this study had no such medical history that might aggravate the dangerous conditions caused by continuous cold compress. During the cold compress process, a dedicated person was arranged to regularly observe the local skin condition of the patients. If any abnormal reactions such as pale skin, cyanosis, numbness, or increased pain occurred, the cold compress was immediately stopped and corresponding treatment measures were taken.

To ensure the stability and operability of the cold compress effect, in this study, medical ice packs were uniformly used as the cold compress material.

#### 2.4.7 Endpoints

The endpoints were evaluated immediately, and at 1.5, 3, 4.5, and 6 h postoperatively. The main outcome measures included pain intensity, facial swelling, maximum mouth opening, and wound hemorrhage.

### 2.5 Sub-study 4: study of duration of cold compress application

#### 2.5.1 Study design

This was a prospective randomized controlled clinical trial aimed at optimizing the duration of cold compress application after mandibular impacted third molar extraction.

#### 2.5.2 Sample size calculation

A power analysis using the G*Power 3.1.9.7 software was conducted. Under the set *α* value of 0.05 and a 90% power analysis condition, each group required 20 patients as the sample size.

#### 2.5.3 Randomization

A group randomization design was adopted. The random allocation sequence was generated by a computer. The confidentiality of the allocation was ensured through measures such as consecutive numbering, sealing, and opaque envelopes. After meeting the inclusion criteria, 80 patients were randomly divided equally into Group A, Group B, Group C and control group.

#### 2.5.4 Inclusion and exclusion criteria

The inclusion and exclusion criteria were the same as those in Sub-study 3.

#### 2.5.5 Surgical methods

The surgical methods were identical to those described in Sub-study 3.

#### 2.5.6 Intervention protocols

Control group: No cold compress applied.
Group A: Continuous cold compress for 6 h within postoperative D1.
Group B: Continuous cold compress for 6 h daily across D1–D2.
Group C: Continuous cold compress for 6 h daily across D1–D3.

All intervention subgroups initiated cold compress immediately postoperatively. Patients returned for follow-up every other day, with scheduled visits timed to match the original extraction appointment to minimize circadian variability.

#### 2.5.7 Endpoints

The endpoints were assessed on postoperative days D1-D3 for pain intensity, facial swelling, maximum mouth opening, and wound hemorrhage.

### 2.6 Outcome measures (common to Sub-study 3 and sub-study 4)

#### 2.6.1 Pain assessment (VAS)

Visual Analog Scale (VAS)/Numerical Rating Scale (NRS) (18) have good reliability and validity. The Cronbach reliability coefficient of VAS and NRS was 0.845 and 0.830, respectively (19, 20): 0: No pain; 1–3: Mild pain; 4–6: Moderate pain; 7–10: Severe pain.

#### 2.6.2 Facial swelling measurement

Facial swelling percentage was calculated using the formula: Facial swelling (%) = Post-compression measurement—Baseline measurement/Baseline measurement × 100% (21). Baseline measurement: Average of two facial distances (cm): Mandibular angle to lateral canthus and Oral commissure to earlobe. Swelling Grading: Grade 0: ≤3%; Grade I: 3%–6%;Grade II: 6%–12%;Grade III: >12%.

#### 2.6.3 Maximum mouth opening (three-finger measurement method)

Normal: 3.5–4.5 cm; Grade I (Mild trismus): 2–2.5 cm; Grade II (Moderate trismus): 1–2 cm; Grade III (Severe trismus): <1 cm; Grade IV (Complete trismus): Lockjaw (22).

#### 2.6.4 Hemorrhage grading

Grade 0: No bleeding; Grade 1: Pink-tinged saliva; Grade 2: Bright red saliva; Grade 3: Frank blood or clots (23).

### 2.7 Statistical analysis

Data were analyzed using SPSS 26.0. Continuous variables are expressed as mean ± standard deviation (Mean ± SD), with intergroup comparisons performed using Student's *t*-test. Categorical variables are presented as frequencies (n), and between-group differences were analyzed via analysis of variance (ANOVA) with *post hoc* Bonferroni correction. Statistical significance was set at *P* < 0.05.

## 3 Results

### 3.1 Results of sub-study 1

As shown in Table 1, we performed the questionnaire survey of patient basic information.

**Table 1:** Survey of patient basic information.

Items	Numbers and percentage
Age (years)	29.2 ± 5.0
Sex	
Male	35 (58.3)
Female	25 (41.7)
Action of postoperative cold compress	
Within 3 h	47 (78.3)
More than 3 h	13 (21.6)
Cold compress material	
Ice pack	58 (96.7)
Other types of ice packs	2 (3.3)
Cold compress method	
Continuous cold compress	23 (38.3)
Intermittent cold compress	37 (61.7)
Whether frostbite has occurred	
Yes	0 (0.0)
No	60 (100.0)

### 3.2 Results of sub-study 2

**Figure 1:**
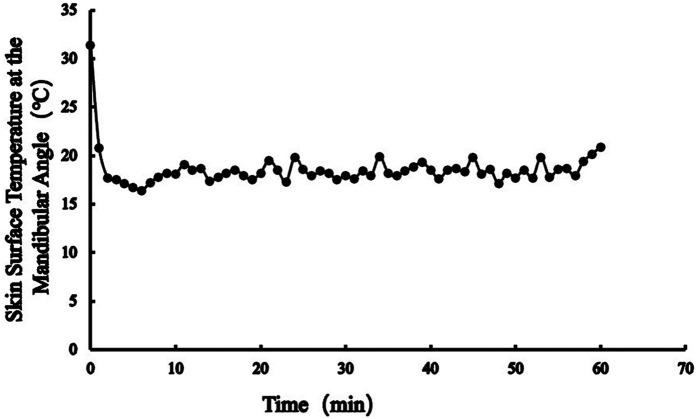
Continue cold compress for 1 h, skin surface temperature at the mandibular angle.

**Figure 2:**
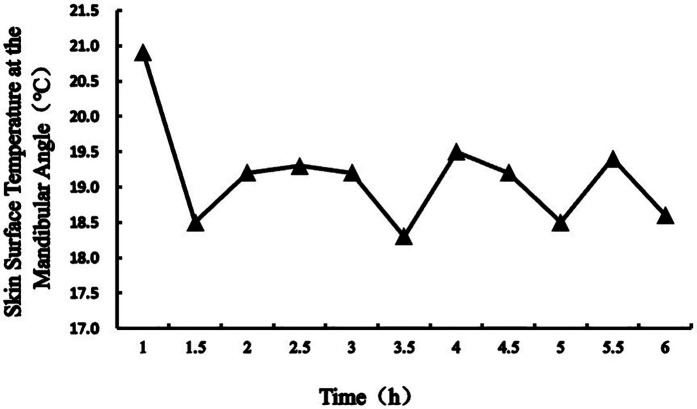
Continue cold compress for 6 h, skin surface temperature at the mandibular angle.

Figure 1 illustrated the skin surface temperature at the mandibular angle during the first hour of continuous cold compress application, while Figure 2 demonstrated the temperature profile over the 6 h duration of continuous therapy.

### 3.3 Results of sub-study 3

#### 3.3.1 General information

**Figure 3:**
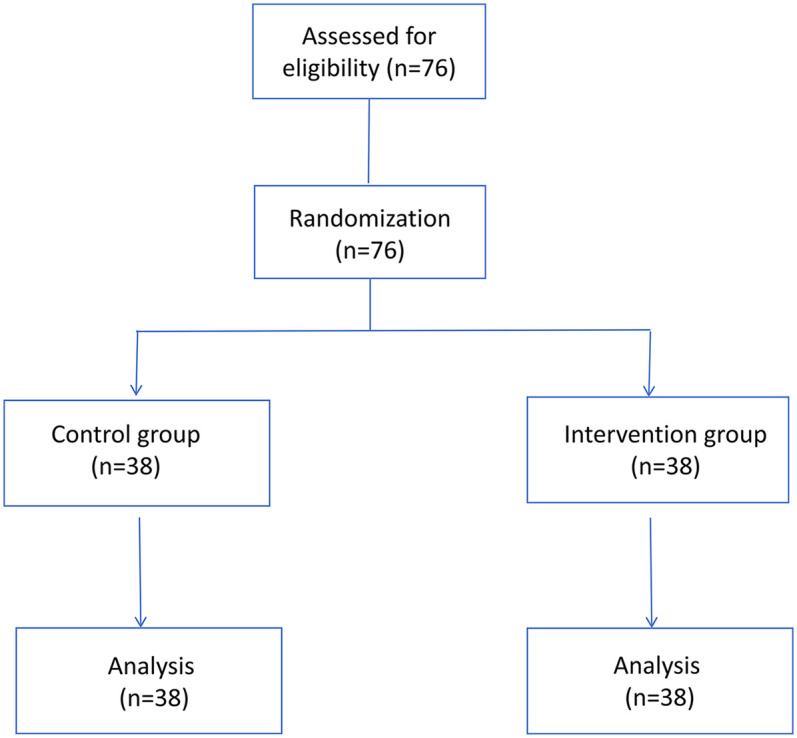
CONSORT diagrams for Sub-study 3.

The CONSORT diagram was shown in Figure 3. There were no significant differences in gender, age, duration of surgery and number of mandibular third molars extracted between the two groups (*P* > 0.05, Table 2), indicating the general information of the two groups were comparable.

**Table 2:** Baseline data of patients between the two groups in Sub-study 3.

Items	Control group (*n* = 38)	Intervention group (*n* = 38)	*P* value
Gender			0.638
Male	14 (36.84)	16 (42.11)	
Female	24 (63.16)	22 (57.89)	
Mean age (years)	29.0 ± 5.4	29.8 ± 5.4	0.642
Duration of surgery (min)	18.1 ± 2.4	18.4 ± 2.5	0.700
Number of mandibular third molars extracted			0.613
One	10 (26.32)	12 (31.58)	
Two	28 (73.68)	26 (68.42)	

#### 3.3.2 Impact of cold compress modalities on postoperative complications

As shown in Table 3, there were no significant differences in VAS scores between the two groups at pre-intervention and 1.5 h, 3 h and 4.5 h post-intervention (*P* > 0.05). However, the VAS scores of the intervention group were lower than those of the control group 6 h post-intervention (*P* < 0.001).

**Table 3:** Comparison of the degree of local pain at different time nodes of cold compress 6 h for two groups of patients.

Times	Control group (*n* = 38)	Intervention group (*n* = 38)	*P* value	Confidence intervals
Before surgery	1.25 ± 0.13	1.27 ± 0.15	0.536	−0.044–0.084
1.5 h after surgery	1.30 ± 0.15	1.29 ± 0.14	0.764	−0.076–0.056
3 h after surgery	1.38 ± 0.16	1.34 ± 0.14	0.249	−0.108–0.028
4.5 h after surgery	2.83 ± 0.30	2.75 ± 0.28	0.233	−0.212–0.052
6 h after surgery	3.86 ± 0.42	3.54 ± 0.36	<0.001	−0.498–0.141

As shown in Table 4, there was no significant difference in the frequency of facial swelling between the two groups at pre-intervention, 1.5 h, 3 h, 4.5 h, and 6 h post-intervention (*P* > 0.05).

**Table 4:** The frequency of I° facial swelling within 6 h of cold compress intervention in two groups of patients.

Times	Control group (*n* = 38)	Intervention group (*n* = 38)	*P* value	Relative risk value
Before surgery	0 (0.00)	0 (0.00)	/	/
1.5 h after surgery	2 (5.26)	1 (2.63)	0.555	2.000
3 h after surgery	2 (5.26)	1 (2.63)	0.555	2.000
4.5 h after surgery	3 (7.89)	2 (5.26)	0.643	1.500
6 h after surgery	5 (13.16)	3 (7.89)	0.185	1.714

There was one patient with limited mouth opening in the intervention group 6 h post-intervention.

As shown in Table 5, there was no significant difference in wound bleeding between the two groups 1.5 h, 3 h and 4.5 h post-intervention (*P* > 0.05). However, the frequency of patients with grade I wound bleeding in the intervention group was lower than that in the control group 6 h post-intervention (*P* = 0.047).

**Table 5:** The frequency of patients with grade I wound bleeding in two groups.

Times	Control group (*n* = 38)	Intervention group (*n* = 38)	*P* value	Relative risk value
1.5 h after surgery	20 (52.63)	18 (47.37)	0.646	1.111
3 h after surgery	17 (44.74)	13 (34.21)	0.347	1.307
4.5 h after surgery	13 (34.21)	7 (18.42)	0.118	1.857
6 h after surgery	6 (15.79)	1 (2.63)	0.047	6.000

### 3.4 Results of sub-study 4

#### 3.4.1 General information

**Figure 4:**
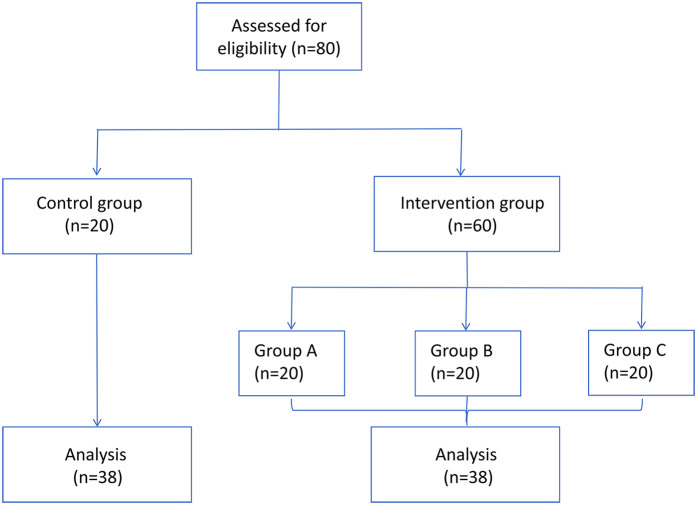
CONSORT diagrams for Sub-study 4.

The CONSORT diagram was shown in Figure 4. There were no significant differences in gender, age, duration of surgery and number of mandibular third molars extracted among the four groups (*P* > 0.05, Table 6), indicating the general information of the four groups were comparable.

**Table 6:** Baseline data of patients among the four groups in Sub-study 4.

Items	Control group (*n* = 20)	Group A (*n* = 20)	Group B (*n* = 20)	Group C (*n* = 20)	*P* value
Gender					0.628
Male	12 (60.00)	9 (45.00)	12 (60.00)	10 (50.00)	
Female	8 (40.00)	11 (55.00)	8 (40.00)	10 (50.00)	
Mean age (years)	29.1 ± 5.3	30.1 ± 5.0	30.3 ± 6.4	30.3 ± 5.9	0.892
Duration of surgery (min)	18.2 ± 2.4	18.6 ± 2.5	18.7 ± 2.6	18.4 ± 2.3	0.958
Number of mandibular third molars extracted					0.933
One	8 (40.00)	9 (45.00)	8 (40.00)	9 (45.00)	
Two	12 (60.00)	11 (55.00)	12 (60.00)	11 (55.00)	

#### 3.4.2 Impact of cold compress duration on postoperative complications

As shown in Tables 7–9, the VAS scores of the experimental subgroups were lower than those of the control group at postoperative Day 1 (*P* = 0.01). However, there were no significant differences in the VAS scores between the experimental subgroups and control group at postoperative Day 2 and Day 3 (*P* > 0.05).

**Table 7:** Comparison of local pain, frequency of II° facial swelling, frequency of II° limited opening of mouth and frequency of grade I wound bleeding between the two groups at postoperative Day 1.

Items	Control group (*n* = 20)	Intervention group (*n* = 60)	*P* value	Confidence intervals or relative risk value
VAS score (points)	3.78 ± 0.43	3.56 ± 0.36	0.010	−0.387–0.052
Frequency of II° facial swelling	1 (2.63)	0 (0.00)	0.206	/
Frequency of II° limited opening of mouth	3 (7.89)	1 (1.67)	0.129	4.736
Frequency of grade I wound bleeding	5 (7.89)	0 (0.00)	0.003	/

**Table 8:** Comparison of local pain, frequency of II° facial swelling, frequency of II° limited opening of mouth and frequency of grade I wound bleeding between the two groups at postoperative Day 2.

Items	Control group (*n* = 20)	Intervention group (*n* = 60)	*P* value	Confidence intervals or relative risk value
VAS score (points)	3.54 ± 0.35	3.46 ± 0.34	0.268	−0.000–0.062
Frequency of II° facial swelling	7 (18.42)	4 (6.67)	0.072	2.763
Frequency of II° limited opening of mouth	11 (28.95)	4 (6.67)	0.002	4.342
Frequency of grade I wound bleeding	1 (13.16)	0 (0.00)	0.206	/

**Table 9:** Comparison of local pain, frequency of II° facial swelling, frequency of II° limited opening of mouth and frequency of grade I wound bleeding between the two groups at postoperative Day 3.

Items	Control group (*n* = 20)	Intervention group (*n* = 60)	*P* value	Confidence intervals or relative risk value
VAS score (points)	2.28 ± 0.23	2.21 ± 0.22	0.139	−0.163–0.023
Frequency of II° facial swelling	16 (42.11)	8 (13.33)	0.001	3.157
Frequency of II° limited opening of mouth	5 (13.16)	2 (3.33)	0.065	3.947
Frequency of grade I wound bleeding	0 (0.00)	0 (0.00)	/	/

There was no significant difference in the frequency of facial swelling between the experimental subgroups and control group at postoperative Day 1 and Day 2. However, compared with the control group, the frequency of facial swelling in the experimental subgroups was lower at postoperative Day 3 (*P* = 0.001).

There was no significant difference in the frequency of limited mouth opening between the experimental subgroups and control group at postoperative Day 1 and Day 3. However, compared with the control group, the frequency of limited mouth opening in the experimental subgroups was lower at postoperative Day 2 (*P* = 0.002).

Compared with the control group, the frequency of patients with grade I wound bleeding in the experimental subgroups was lower at postoperative Day 1 (*P* = 0.003). However, there was no significant difference in the frequency of patients with grade I wound bleeding between the experimental subgroups and control group at postoperative Day 2 and Day 3 (*P* > 0.05).

## 4 Discussion

### 4.1 Questionnaire survey

The survey results indicated that 78.3% of patients applied cold compress for <3 h after mandibular impacted third molar extraction, with only 21.7% adhering to ≥3 h. This highlights suboptimal patient compliance with recommended cold compress protocols.

### 4.2 Pilot study

Temperature monitoring revealed fluctuations in skin surface temperature at the mandibular angle (17–21 °C) during cold compress application. Cyclical cooling-rewarming phenomena were observed at 1.5, 3.5, and 5 h, consistent with the physiological “vasoconstriction-dilation-constriction” mechanism triggered by cryotherapy (16, 24). Although this mechanism was activated during the 6 h cold compress protocol, temperatures remained above the frostbite threshold (critical range: 10–15 °C), and no adverse reactions (e.g., numbness, erythema) were reported. These findings confirm the safety profile of a 100 mL pure-water ice pack wrapped in a towel for continuous 6 h cold compress application at the mandibular angle.

Given that postoperative pain typically peaks within 6–8 h after extraction (25), yet most patients exhibit poor compliance beyond 3 h, the 6 h timeframe was selected to systematically evaluate the efficacy of different cold compress regimens (continuous vs. intermittent) and durations in mitigating postoperative complications.

### 4.3 Randomized controlled trial

#### 4.3.1 Impact of cold compress modalities on postoperative complications

This study demonstrated that continuous cold compress application within the first 6 postoperative hours significantly outperformed intermittent protocols in analgesia, edema reduction, trismus alleviation, and hemorrhage control. The underlying mechanisms are elucidated through three key pathways:

##### 4.3.1.1 Stability of hemodynamic regulation

Intermittent cold compress therapy induces reactive vasodilation during off intervals due to temperature rebound, exacerbating secondary tissue exudation and inflammatory mediator release (e.g., bradykinin) (24), which aggravates swelling and pain. In contrast, continuous cold compress maintains sustained vasoconstriction, reducing endothelial intercellular gap formation and thereby inhibiting the extravasation of nociceptive factors (26).

##### 4.3.1.2 Profound suppression of inflammatory pathways

Hypothermia significantly downregulates cyclooxygenase-2 (COX-2) activity, suppressing prostaglandin E2 (PGE2) synthesis (27) and blocking nociceptive signaling. However, intermittent protocols allow localized temperatures to intermittently exceed 21 °C, permitting partial COX-2 reactivation and diminished anti-inflammatory efficacy. The markedly lower VAS scores observed in the continuous cold compress group align with this mechanistic framework.

##### 4.3.1.3 Cumulative metabolic suppression

Prolonged hypothermia induces cumulative metabolic inhibition. Studies indicate that each 1 °C reduction in tissue temperature decreases cellular metabolic activity by 6%–7% (28), delaying neutrophil chemotaxis and reducing edema risk. The significantly attenuated swelling in the continuous cold compress group at 6 h postoperatively validates this pathway.

#### 4.3.2 Impact of cold compress duration on postoperative complications

##### 4.3.2.1 Acute phase (0–24 h)

Continuous cold compress application within postoperative D1 is critical for hemorrhage control and acute pain management. Hypothermia enhances platelet aggregation, significantly reducing hemorrhage incidence, while concurrently suppressing prostaglandin E2 (PGE2) biosynthesis, decreasing tissue metabolic activity, and slowing nociceptive nerve conduction velocity (27). These mechanisms collectively attenuate postoperative pain intensity. In this study, Group A (6 h continuous cold compress within D1) exhibited significantly lower hemorrhage rates (*P* < 0.01) and VAS scores (*P* < 0.05) compared to controls, validating the clinical utility of acute-phase cold compress therapy. Consistently, it has been reported that cryotherapy methods improve pain and function of patients after total knee arthroplasty (29). Benchahong et al. suggested that cold therapy both before and after amniocentesis procedure is most effective in pain reduction (30).

##### 4.3.2.2 Subacute phase (D1–D3)

Prolonged cold compress application through D3 significantly improves edema resolution and trismus. The 48–72 h postoperative period corresponds to peak inflammatory cell infiltration, during which macrophages release interleukin-6 (IL-6) and tumor necrosis factor-α (TNF-α) via the TLR4/NF-κB pathway. Sustained hypothermia inhibits heat shock protein 70 (HSP70) activation, thereby blocking NF-κB nuclear translocation and reducing IL-6 levels (31). Furthermore, localized cooling mitigates intramuscular spindle pressure elevation in the masseter muscle, dampening *γ*-motor neuron excitability (32), which enhances mouth opening capacity.

#### 4.3.3 Clinical practice recommendations

Based on the findings, a staged cold compress protocol is proposed: Phase I (0–6 h postoperatively): Continuous cold compress application, prioritizing acute hemorrhage control and pain mitigation; Phase II (D1–D3): Daily 6 h continuous cold compress sessions to suppress inflammatory edema and improve trismus resolution.

#### 4.3.4 Comparison with alternative and emerging postoperative management techniques

In recent years, several new postoperative management techniques have emerged in tooth extraction surgery, including platelet-rich fibrin (PRF), ozone therapy, low-level laser therapy (LLLT) and kinesiology taping (33–36). PRF is known for its regenerative and anti-inflammatory properties, and thus increasingly being used to reduce postoperative pain, swelling, and symptoms of alveolar osteitis (37). Compared with PRF, cold compresses do not require invasive procedures, are inexpensive, and can be applied immediately. However, PRF achieves long-term effects through biological repair, while cold compresses only provide short-term symptom relief. The two can complement each other (for example, early cold compress after surgery to control acute symptoms, and later filling with PRF to promote healing). Ozone therapy has shown promise in minimizing postoperative discomfort and promoting healing due to its antimicrobial and oxygenating effects (38). Compared with ozone therapy, cold compresses have no odor and no injection risks, and can be operated independently; ozone therapy, on the other hand, requires professional training from medical staff and has a low equipment penetration rate. However, ozone therapy achieves comprehensive effects by improving the micro-environment, while cold compresses only address the symptoms. The two can be used together (for example, ozone rinse followed by cold compress to reduce exudation). LLLT inhibits the expression of COX-2 through photobiological regulation, reduces the synthesis of PGE2, and simultaneously promotes mitochondrial ATP production, thereby alleviating muscle spasms and pain (39). Compared with LLLT, cold compresses are extremely cost-effective and can be applied immediately; LLLT requires specialized equipment and its effectiveness is influenced by the operator's experience. However, LLLT achieves deep tissue repair through biological stimulation, while cold compresses only act on the surface tissues. The two can work together (for example, after cold compresses control acute symptoms, LLLT promotes functional recovery). Kinesiology taping is an innovative non-invasive therapy that has been proven to alleviate swelling after oral surgery and facilitate the drainage of lymph fluid (40). Compared with kinesiology taping, cold compresses do not require special training and can cover large areas; kinesiology taping, on the other hand, need to be custom-cut and may cause skin allergies. However, kinesiology taping improves lymphatic circulation through mechanical action, while cold compress works through thermodynamic effects. The two can be combined (for example, applying the kinesiology taping after cold compressing to prolong the time for reducing swelling). By comparison, it can be seen that the “6 h continuous cold compress protocol” proposed in this study has irreplaceable advantages in terms of accessibility, cost and short-term efficacy, and is particularly suitable for promotion in grassroots medical institutions. At the same time, the research results provide a theoretical basis for the multimodal postoperative management of “cold compress + emerging technologies”, promoting the clinical transition from “single treatment” to “integrative medicine” mode. In the future, a head-to-head randomized controlled trial needs to be conducted to clarify the optimal combination sequence and timing of different technologies, in order to further optimize the nursing path.

## 5 Limitations

This study has several limitations: Pressure parameters influencing tissue osmotic gradients were not evaluated; Limited sample size necessitates multicenter validation; Long-term complications (e.g., alveolar osteitis) were not assessed. Future studies should incorporate infrared thermography for real-time deep tissue temperature monitoring and develop intelligent cold compress devices to achieve precise modulation of temperature, duration, and pressure gradients.

## 6 Conclusion

Our research indicates that, within the first three days after mandibular impacted third molar extraction, applying cold compress for a continuous 6 h each day can significantly reduce complications and achieve the best clinical outcome.

## Data Availability

The original contributions presented in the study are included in the article/Supplementary Material, further inquiries can be directed to the corresponding author.
