# Supplementary material for: Clinical study on the effects of different cold compress methods and durations on postoperative complications following mandibular impacted third molar extraction
Source: Front Surg. 2025 Nov 24;12:1627139. doi: 10.3389/fsurg.2025.1627139 (PMC12683523; doi:10.3389/fsurg.2025.1627139)
Supplement: Supplementary file 1 [file Table1.docx]

**Patient Basic Information Questionnaire**

1. Age:
2. Sex:

Male ( ) Female ( )

1. Action of postoperative cold compress

Within 3 hours ( ) More than 3 hours ( )

1. Cold compress material

Ice pack ( ) Other types of ice packs ( )

1. Cold compress method

Continuous cold compress ( ) Intermittent cold compress ( )

1. Whether frostbite has occurred

Yes ( ) No ( )
